# XBP‐1s transcriptional targets in the endoplasmic reticulum unfolded protein response ameliorate tauopathy

**DOI:** 10.1002/alz.088582

**Published:** 2025-01-03

**Authors:** Brian C. Kraemer, Marina Han

**Affiliations:** ^1^ University of Washington School of Medicine, Seattle, WA USA; ^2^ University of Washington, Seattle, WA USA

## Abstract

**Background:**

Protein homeostasis (proteostasis) mechanisms fail with aging and disease, promoting toxic protein accumulation. Neurons are particularly vulnerable to proteostatic disruption leading to aging related neurodegeneration. Abnormal activation of the endoplasmic reticulum unfolded protein response (UPR^ER^) is implicated in tauopathies, a group of neurodegenerative diseases characterized by pathological accumulation of the microtubule‐associated protein tau. Previous work showed neuronal overexpression of IRE1a branch UPR^ER^ transcription factor XBP‐1s suppresses tauopathy in *C. elegans*.

**Methods:**

We conducted RNA sequencing of mRNAs to analyze the transcriptome of xbp‐1s and tau transgenic animals to identify genes required for XBP‐1s mediate protection from tauopathy.

**Results:**

Transcriptomic analysis of XBP‐1s animals showed upregulation of the following genes with human homologs: *csp‐1, F42G8.7, F41E7.6, Y19D10A.16, C01B4.6, dnj‐28, hsp‐4, ckb‐2, mct‐2, lipl‐3*, and *eol‐1*. Surprisingly, each one of these genes is required for *xbp‐1s*‐mediated suppression of tauopathy, suggesting that XBP‐1s activates a broad and non‐redundant network of cellular mechanisms to reduce tau pathology. Of these, we examined the critical UPR^ER^ regulator BiP/hsp‐4, the ER resident HSP70 homolog. *Hsp‐4* loss of function, but not loss of the cognate ER resident DNAJ protein *dnj‐28*, eliminates *xbp‐1s*‐mediated suppression of tauopathy. While *hsp‐4* loss of function exacerbates tau‐induced behavioral deficits, tau protein level and phosphorylation are unaffected. High level overexpression of *hsp‐4* exacerbates tau‐induced behavioral deficits and protein accumulation, while moderated *hsp‐4* overexpression ameliorates this phenotype. Furthermore, caspases also appear to play an important role downstream of XBP‐1s: both the XBP‐1s target *csp‐1* and its non‐XBP‐1s target gene family member *ced‐3* are required for *xbp‐1s*‐mediated suppression of tauopathy.

**Conclusions:**

We present a dataset illuminating the mechanism of *xbp‐1s*‐mediated suppression of tauopathy involving a suite of diverse transcriptional targets.

